# Residual force enhancement following shortening is speed-dependent

**DOI:** 10.1038/srep21513

**Published:** 2016-02-12

**Authors:** Rafael Fortuna, Geoffrey A. Power, Esther Mende, Wolfgang Seiberl, Walter Herzog

**Affiliations:** 1 Human Performance Laboratory, Faculty of Kinesiology, University of Calgary, Calgary, Canada; 2 Department of Human Health and Nutritional Sciences, College of Biological Sciences, University of Guelph, Guelph, Ontario, Canada; 3 Department of Biomechanics in Sports, Faculty of Sport and Health Sciences, Technische Universität München, Munich, Germany

## Abstract

The steady-state isometric force following active muscle shortening or lengthening is smaller (force depression; FD) or greater (residual force enhancement; RFE) than a purely isometric contraction at the corresponding length. The mechanisms underlying these phenomena are not explained within the context of the cross-bridge theory and are rarely studied in concert. Previous studies have shown RFE to be speed-independent. In the present study, we investigated if RFE preceded by active shortening is time-dependent by electrically evoking RFE in the human adductor pollicis muscle. The results shown that a slow stretch following FD fully re-established RFE compared to higher speeds of stretch. The mechanism(s) responsible for the recovery of RFE following a preceding shortening contraction (FD) might be associated with the recovery of cross-bridge based force and/or the re-engagement of a passive structural element (titin). Voluntary interaction with one’s environment involves highly coordinated shortening and lengthening muscle contractions. Therefore comprehending these history-dependent muscle properties in the context of movement control is paramount in understanding the behavior of *in vivo* motor control.

Muscle force production is history-dependent^1^,^2^,^3^,^4^,^5^. Therefore, the steady-state isometric force following a shortening or lengthening contraction is smaller (force depression; FD) or greater (residual force enhancement; RFE) than a purely isometric contraction for the same level of activation and at corresponding muscle length^1^,^3^. These history-dependent properties have been well-documented across all structural levels of muscle in a variety of preparations^4^,^6^,^7^,^8^,^9^,^10^,^11^,^12^. However, despite extensive research over the last 50 years, the mechanisms underlying these phenomena are still not fully understood.

Investigations into the mechanisms underlying FD and RFE are often performed separately, with only a few studies investigating both of these history-dependent properties in concert^11^,^13^,^14^,^15^. During functional movement tasks, skeletal muscles undergo stretch-shortening and shortening-stretch cycles on a regular basis. Thus, determining how shortening preceding a stretch affects muscle function is essential for understanding human movement control and basic mechanisms of muscle contraction. In a study of frog single muscle fibers, Rassier *et al*. (2004) observed that an active stretch immediately following shortening of varying lengths caused the force to be lower compared to the same stretch not preceded by shortening. Similarly, Herzog & Leonard (2000) showed that active shortening preceding stretch eliminated force enhancement in the cat soleus entirely under some conditions. These findings indicate that active muscle shortening prior to stretch decreases RFE in a shortening amplitude-dependent manner. In contrast, Edman *et al*. (1982) found that shortening preceding stretching did not affect RFE in single frog muscle fibers. However, and in contrast to the other studies, Edman’s shortening and stretch conditions were separated by a one second break.

Summarizing, it appears that active muscle shortening immediately preceding stretch reduces RFE, while increasing time intervals between the shortening and stretch conditions re-establishes the full RFE once the interval reaches about 1 s. These findings indicate that active shortening produces FD that reduces the subsequent RFE, but that the effect of the active shortening disappears with time. However, this result, shown in single frog fibres and isolated cat soleus muscles, has never been confirmed in human skeletal muscles. Therefore, the first purpose of this paper was to determine if a time interval between active shortening and the subsequent active stretching helps to re-establish the full RFE seen for pure stretch conditions not preceded by shortening.

Furthermore, it is well accepted that RFE is independent of the speed of active muscle stretching^1^,^3^. However, if a time interval between active shortening and the subsequent stretch re-establishes the full RFE in a time-dependent manner, then it might be possible that a stretch following shortening at a slow speed (which takes a lot of time) might produce a greater RFE than a stretch at a fast speed (which takes little time). In other words, RFE might depend on the speed of stretch, if muscle stretching is preceded by active shortening. However, such a speed dependence of RFE has never been observed under (any) conditions, and the specific case here has not been studied. If indeed RFE was stretch-speed dependent when preceded by active shortening, then this result might provide crucial information about the mechanisms of FD and RFE. Therefore, the second purpose of this study was to determine if RFE depends on the speed of stretching when stretching is preceded by active muscle shortening. We hypothesized that active shortening preceding stretch reduces the RFE, and that this effect is abolished if the time between shortening and stretch exceeds a critical value or if the stretch is made at a sufficiently slow speed.

## Methods

In order to address the two purposes of this study, two distinct experiments were performed. In the first set of experiments, active muscle shortening was separated from the active stretch conditions by varying time intervals. In the second set of experiments, the stretch conditions followed the active shortening without time delay but were performed at different speeds.

### Participants

Twelve healthy male participants (age 28.5 ± 5.8 years; height 180 ± 6 cm; weight 76 ± 6 kg) participated in protocol 1, while protocol 2 had the same number of subjects with similar group characteristics (age: 27.2 ± 3.8 years; height: 180 ± 10 cm; weight: 75 ± 7 kg). All participants were well familiar with the procedures and neuromuscular techniques used. They were free of neuromuscular disorders or injury to the left hand. This study was approved by The Conjoint Ethics Committee of the University of Calgary and conformed to the Declaration of Helsinki. Informed written consent was obtained from all participants prior to testing.

### Experimental Setup

Thumb adduction forces and carpometacarpal angular displacements were measured using a custom-designed dynamometer^7^,^16^. The left hand was immobilized with a reusable clinical cast (Ezeform, Rehabilitation Division, Smith & Nephew Inc., Germantown, WI, USA) and was secured with two velcro straps, restricting movement of the wrist and fingers except for the thumb. Participants sat on an adjustable chair with the shoulder slightly abducted and the elbow flexed at 90°. A rotary stepper motor (Model TS42BP10, Parker Hannifin Corp., Cleveland, OH, USA) was connected to an aluminum rod (1.5 cm diameter and 15 cm long) via gears (1:4 gear ratio). The other end of the rod was attached to an auxiliary piece for thumb placement and fixation. The thumb pressed on the auxiliary piece which was in line with the direction of force measurement obtained through two pairs of calibrated strain gauges (Model CEA-06–125UN-350, Measurement Group, Inc. Raleigh, NC, USA). The forearm was slightly supinated relative to the thumb placed on the auxiliary piece, thus thumb movement was guided toward the third finger. Thumb angle was measured using an analogue encoder (Series 03 rotary transducer, Hohner Corp., UK). A digital controller (Model Gemini GT6-L8 Digital stepper driver/controller, Parker Hannifin Corp., Cleveland, OH, USA) controlled the rod. A 0° reference angle was defined for each subject as the highest degree of thumb adduction possible before the dynamometer arm came in contact with the cast. Thumb angles increased with abduction, ranging from 0–30°.

### Electrical stimulation

Two self-adhering Ag-AgCl surface electrodes (2 × 3 cm) were placed over the ulnar nerve to electrically stimulate the adductor pollicis muscle. The cathode was placed approximately 2 cm proximal to the pisiform bone on the medial wrist, and the anode was placed 2 cm proximal to the cathode. A computer-triggered stimulator (model DS7AH, Digitimer, Welwyn Garden City, Hertfordshire, UK) was used to increase current until maximum single twitch force peak was reached (single 100 μs square-wave pulses). These settings were used to assess voluntary activation (VA) during maximum voluntary contraction (MVC) using the interpolated twitch technique (Gandevia, 2001) at a carpometacarpal angle of 30°. All subsequent contractions were electrically evoked. Tetanic electrical stimulation for all contractions was performed with an identical setup. The amperage of the stimulator was increased (50 Hz; square wave pulses with 100 μs pulse width, 400 V) until force reached 50–60% of the participant’s MVC, and was kept constant throughout the experimental period.

### Experimental Protocols 1 and 2

**Figure 1:**
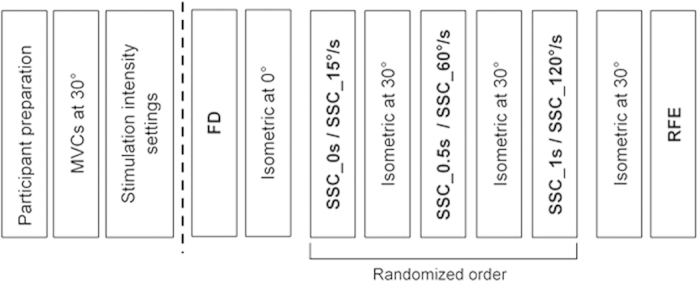
Experimental design for protocols 1 & 2. Following participant preparation, MVC was measured at a 30° thumb abduction angle. Stimulation intensity was set to 50–60% of MVC. Protocol 1 & 2 started with FD followed by the appropriate isometric test. Next, a randomized order of SSCs with either a (i) varying time interval between shortening and stretching (SSC_0 s, SSC_0.5 s, SSC_1 s; protocol 1) or (ii) varying stretch speeds (SSC_15°/s, SSC_60°/s, SSC_120°/s; protocol 2), followed by the corresponding isometric test. Last, protocol 1 & 2 tested RFE with the corresponding isometric test.

Participants were encouraged verbally to produce a 3 s MVC and only participants with VA >95% were used for further testing. The protocol consisted of a FD test, followed by 3 separate shortening-stretch cycles, and finally a single test for RFE. Following each shortening-stretch experimental test, the initial isometric test was repeated to assess possible effects of fatigue (Fig. 1).

### Force depression test (FD)

The muscle was pre-activated and held isometrically for 1 s at 30°, this was followed by a 0.5 s of shortening contraction over a 30° joint excursion at an angular velocity of 60°/s. The muscle was then held isometrically at 0° for 5.5 s. Next, a purely isometric reference contraction was performed at 0° for 7 s.

**Figure 2:**
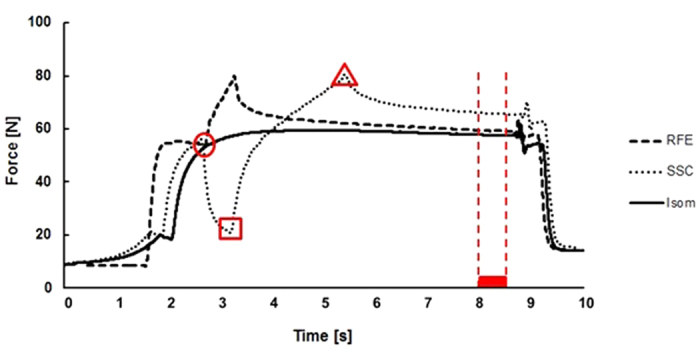
Exemplar raw force trace for a participant performing an isometric reference contraction at 30° thumb abduction (solid line, Isom), a pure stretch-induced residual force enhancement (dashed line, RFE) and a shortening-stretch cycle in which stretching follors the shortening phase immediately at a 15°/s stretch velocity (dotted line, SSC). The average force (mean 200 ms) before shortening (b-SHO, •), the minimum force at the end of shortening shortening (e-SHO, ■), the maximum force at the end of stretching (e-STR, ▲), and the average isometric steady-state force (500 ms) prior to muscle deactivation.

The beginning of the shortening-stretch cycle (SSC) tests was similar to the FD test (Fig. 2). For the SSC tests, the shortening phase was kept identical in magnitude and speed, while the stretch phase was either separated from the shortening phase by varying time intervals (protocol 1) or was performed at different stretch speeds (protocol 2).

### Protocol 1

Following shortening (30° to 0° at 60°/s), the muscle was then either; immediately stretched back to 30° at 60°/s (SSC_0 s), held isometrically for 0.5 s (SSC_0.5 s) then stretched back to 30°, or held isometrically for 1 s (SSC_1 s) and then stretched back to 30°. Following stretch, all SSCs (SSC_0 S, SSC_0.5 s, and SSC_1 s) were held isometrically at 30° angle for 5, 4.5, and 4 seconds, respectively. The order of time intervals between the shortening and stretch conditions was randomized and all stretch conditions were performed at a fixed angular speed of 60°/s.

### Protocol 2

The shortening (30° to 0° at 60°/s) was followed immediately by a 30° stretch. The stretch phases were performed at angular speeds of 15°/s (SSC_15°/s), 60°/s (SSC_60°/s), or 120°/s (SSC_120°/s), then held isometrically at 30° for 3.5, 5, and 5.25 s, respectively. The order of the stretch speeds was randomized.

Following the SSCs of protocol 1 and 2, an isometric reference contraction lasting 7 s was performed at a thumb abduction angle of 30°. Then, the RFE was assessed by pre-activating the muscle at a thumb abduction angle of 0° for 1 s, abducting the thumb to 30° at an angular speed of 60°/s, and then holding the muscle isometrically for another 5.5 s.

### Data reduction and analysis

All data were sampled at 1000 Hz and collected via an analog-to-digital converter (PowerLab System 16/35, ADInstruments, Bella Vista, Australia). Force data were filtered (lowpass 10 Hz). Force before shortening (200 ms average; b-SHO), at the end of shortening (minimum force; e-SHO), at the end of stretching (maximum peak force; e-STR), and mean force during a 500 ms time period prior to muscle deactivation were used for statistical analysis (Fig. 2). The mean force values for the SSCs were compared to the corresponding values obtained from the isometric reference contractions. Work during the shortening phase for the FD tests and the SSCs was calculated as the product of mean force during shortening, the length of the lever arm and the thumb displacement (30°).

### Statistical analysis

Data were tested for normality (Kolmogorov-Smirnov test) and a repeated measures ANOVA (non-parametric Friedman analysis) with Bonferroni-Holm post-hoc correction (Students t-tests) was used to identify significant differences between parameters of (mean) forces, work during shortening, and for comparisons of forces obtained in the SSCs and the purely isometric reference contractions. The level of significance was set at α = 0.05. Outliers, defined as force values deviating the respective mean by more than ±2SD, were excluded from statistical analysis.

## Results

### Protocol 1

The mean electrically evoked tetanic isometric force at 30° was 54.4 ± 6.4 N, representing 54.7% of the initial force achieved during MVCs (99.6 ± 18.2 N). Two subjects were excluded from the data analysis due to inconsistent values throughout the protocol.

There was no significant difference in the force achieved before shortening (b-SHO), minimum force achieved at the end of shortening (e-SHO) and the peak force at the end of stretching (e-STR) (Fig. 2), except SSC_1 s showed a significantly lower force at the end of shortening (e-SHO) compared to FD (Table 1). Force at the end of stretching (e-STR) for all SSCs was significantly lower compared to the pure stretch condition (RFE). Additionally, there was no significant difference in the work performed during pure shortening (FD tests) and shortening during the different SSCs (Table 1).

**Figure 3:**
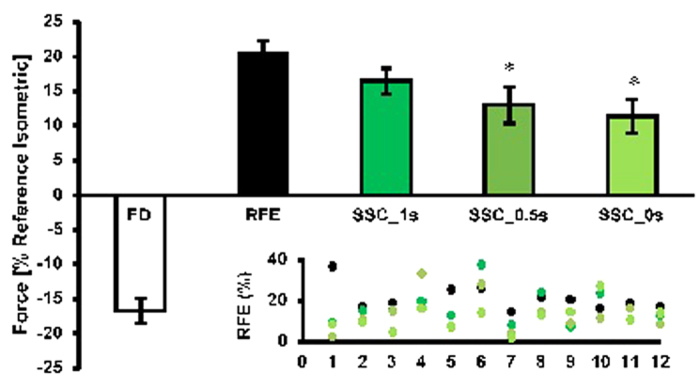
Mean values of force decrease (FD) and force increase (RFE, SSC_1 s, SSC_0.5 s, SSC_0 s) (±SE) normalized to the values of the isometric reference contraction at the corresponding thumb angle for protocol 1. Residual force enhancement (RFE) for the pure stretch condition was 20.6%; whereas RFE for the SSC_0 s and SSC_0.5 s conditions was significantly lower (11.4 & 13.0%) than RFE for the pure stretch conditions. When a 1 s interval (SSC_1 s) was introduced between the shortening and the stretch phase, the effect of shortening on force enhancement were abolished and the forces between the two conditions were similar (16.5 ± 8.5%). *compared to RFE. Inset: scatter plot for each individual subject. Note that the colors of the scatter plot correspond to the colors in the main graph.

The steady-state isometric force was significantly (*p* < 0.05) depressed by 16.7 ± 6.2% following active shortening (FD) compared to the reference isometric contraction at the corresponding length. Force was significantly (*p* < 0.05) increased by 20.6 ± 6.2% following active stretching (RFE) compared to the reference isometric contraction at the corresponding length (Fig. 3). For the shortening-stretch cycles (SSCs), the steady-state isometric force was significantly (p < 0.05) reduced in comparison to pure stretch RFE (20.6 ± 6.2%) when the time interval between shortening and stretching was 0 s and 0.5 s (11.4 ± 6.2% and 13.0 ± 9.2% for SSC_0 s and SSC_0.5 s, respectively). However, when a 1 s time interval (SSC_1 s) was given between shortening and stretching, the values at steady-state (16.5 ± 8.5%) were not significantly different from the corresponding forces for the pure stretch RFE contractions (Fig. 3).

### Protocol 2

In the second protocol, the mean electrically evoked tetanic isometric force at 30° was 54.8 ± 10.2 N, representing 54.5% of the initial force achieved during MVCs (96.5 ± 13.6 N).

There was no statistical difference in the muscle forces before shortening (b-SHO) and at the end of shortening (e-SHO) (Fig. 2) among all SSC and the FD conditions. Forces at the end of stretching (e-STR) were lower for all SSC compared to the pure stretch conditions (RFE; Table 2). Peak force at the end of stretching (e-STR) was greatest for the fastest stretch speed (SSC_120°/s). There was no significant difference in the work performed during shortening for the FD tests and the different SSCs (Table 2).

**Figure 4:**
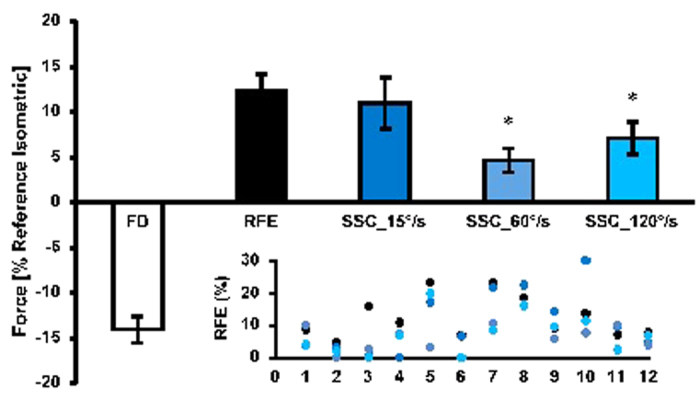
Mean values of force decrease (FD) and force increase (RFE, SSC_15°/s, SSC_60°/s, SSC_120°/s) (±SE) normalized to the values of the isometric reference contractions at the corresponding thumb angle for protocol 2. Residual force enhancement (RFE) for the pure stretch conditions was 12.3%; whereas RFE for the SSC_60°/s and SSC_120°/s conditions was significantly lower (4.7 & 7.1%, respectively) than RFE for the pure stretch conditions. When stretching was performed at 15°/s (SSC_15°/s), the effect of shortening on force enhancement was abolished and the forces between the two conditions were similar 11.0 ± 9.8%). *compared to RFE. Inset: scatter plot for each individual subject. Note that the color scheme in the scatter plot matches that of the main figure.

The steady-state isometric force was significantly (*p* < 0.05) depressed by 14.9 ± 5.0% following active shortening (FD) compared to the isometric reference contraction at the corresponding length. Force was significantly (*p* < 0.05) increased by 12.3 ± 6.4% following active stretching (RFE) compared to the corresponding isometric reference contraction (Fig. 4). Force enhancement following the 60 and 120°/s stretch speeds was significantly (*p* < 0.05) reduced (4.7 ± 4.6% and 7.1 ± 6.2% for SSC_60°/s and SSC_120°/s, respectively) compared to the pure stretch RFE condition (12.3 ± 6.4%). However, when stretching was performed at 15°/s, force enhancement was similar to the pure RFE condition (11.0 ± 9.8% & 12.3 ± 6.4% for SSC_15°/s & RFE, respectively; p > 0.05; Fig. 4).

## Discussion

The present study was designed to investigate the time- and speed-dependence of stretch-induced RFE when active stretch started from a shortening-induced force depressed state. In line with our hypothesis, we show that when an active stretch is performed immediately and 500 ms following shortening the steady-state isometric force is lower than that obtained for pure RFE conditions where a stretch is not preceded by shortening. However, when sufficient time is provided between the shortening and stretch contractions, the steady-state isometric force following stretch becomes independent of the shortening. These findings indicate that the shortening-induced force depression affects the subsequent stretch-induced force enhancement, but that this force depression effect disappears within about 1 s in an intact, *in vivo* human muscle at a physiological temperature. Therefore, whatever the mechanism for RFE is, this mechanism is perturbed immediately following shortening causing a reduced force enhancement, but appears to be fully re-established if there is sufficient time between the active shortening and the active stretch of a muscle.

Despite evidence that RFE after pure stretch is not speed-dependent^1^,^3^,^7^, we found here that RFE indeed depends on the speed of stretch, if muscle stretching is immediately preceded by active muscle shortening: slow speeds of stretch were associated with increased RFE. These findings indicate that the perturbed mechanism for force enhancement following active muscle shortening, can be re-established during active stretching given sufficient time. Therefore, a slow stretch speed (following active muscle shortening), which allows for a long time to re-establish the conditions for producing force enhancement, results in a virtually normal RFE, while for a fast stretch, when there is insufficient time, the effects of active shortening remain present and reduce the amount of RFE.

It has been argued that RFE is caused by increased forces following stretch from the actin-myosin based cross-bridges and/or from increased forces originating in passive structural elements, such as titin^5^,^7^,^17^ Experiments in which the shortening conditions were changed (magnitude and speed of shortening), or in which the activation was changed in the interval between the shortening and stretch conditions, might prove useful in distinguishing between competing mechanisms.

### Transient force values

The force values before shortening (b-SHO) and at the end of shortening (e-SHO) were assessed in order to assure a consistent force output throughout the testing protocol. As one would expect, force before shortening (b-SHO) was similar for all experimental conditions in protocols 1 and 2. Despite the fact that force at the end of shortening (e-SHO) was significantly lower for the SSC_1 s compared to the pure shortening (FD) conditions in protocol 1 (Table 1), force redeveloped to a similar steady-state force as that observed in the pure RFE conditions. Force at the end of stretching (e-STR) for all SSC conditions in both protocols was lower compared to the RFE tests, possibly because active shortening causes a stress-induced inhibition of cross-bridge formation, which may not have been recovered in the subsequent active stretch^2^. Increasing stretching speeds are not associated with increased steady-state isometric forces following stretching under normal conditions^1^,^3^, but they are known to cause increased peak forces at the end of stretching (e-SHO) according to the cross-bridge theory^18^ and the force-velocity properties of skeletal muscles^7^,^19^,^20^,^21^. The speed dependence of force during active muscle lengthening was confirmed in our study as the highest stretch speed (120°/s) produced significantly higher peak forces than the two lower speeds tested here (15°/s and 60°/s). Since all experiments were performed using the same shortening conditions that gave similar results for all conditions up to the end of the shortening phase, all differences in peak forces during stretch and following stretch must be explained with the different conditions (variable pause between shortening and stretch, and variable stretch speeds) following the shortening phase.

### Time-dependent RFE

It has been shown that active shortening preceding a stretch reduces the stretch-induced RFE, but this reduction disappears if shortening and stretch are separated by sufficient time^11^. Our results on *in vivo* human skeletal muscles agree with those found in single intact fibres from frogs. Specifically, we found that the steady-state isometric forces following SSC with 1s pause between shortening and stretch were similar to those obtained for the pure stretch RFE condition, and were greater than the corresponding forces obtained for SSCs with time intervals between shortening and stretching that were shorter than 1 s (SSC_0.5 s, SSC_0 s). These results suggest that the time dependence of the recovery of the full RFE following active muscle shortening is a property that manifests itself on the single fibre and muscle level to a similar degree, and thus might be relevant in every day animal movements.

### Speed-dependent RFE

We showed that the redevelopment of full RFE in protocol 1 depends on the pause given between the active shortening and the subsequent active stretching of the muscle. This result suggests that the re-development of the full RFE following active shortening requires time. It almost appears as if the muscle must “recover” from the FD effect of the active shortening contraction before it can fully deploy its force enhancement properties. In protocol 1, time for recovery was provided by separating the shortening and stretch conditions by a variable pause, and holding the active muscle at a constant length during this pause. In protocol 2, the variable speed of stretch provides for variable amounts of time between the end of active shortening, and the achievement of the final length following muscle stretching. If time is indeed the crucial factor for recovering the full potential for producing RFE in the active stretch conditions, then one would expect RFE to be greater following a slow stretch (more time) compared to a fast stretch (less time). Our results confirmed that a slow stretch speed (15°/s) was associated with a full recovery of the RFE, while for the fast stretch speeds (60 and 120°/s) RFE was reduced compared to the pure stretch condition. Therefore, it seems that RFE preceded by active muscle shortening is stretch speed-dependent; with slow speeds allowing for full force enhancement to occur, while fast speeds result in a decreased RFE.

### Potential contributing mechanisms to the time- and speed-dependence of RFE

Force depression following active shortening is thought to be caused by a stress-induced inhibition of cross-bridge attachments^2^. We speculate that the reduction in RFE following a stretch that is preceded by active shortening is associated, at least in part, with the inhibition of cross-bridges following shortening. Given sufficient time following active muscle shortening, such as for the SSC_1 s and SSC_15°/s conditions, the shortening-induced cross-bridge inhibition may be abolished or may not affect the subsequent stretch-induced RFE. Knowing that FD is long lasting, and thus cross-bridge inhibitions following shortening are maintained as long as the muscle remains activated^1^,^22^,^23^ it appears that a recovery of cross-bridge inhibition is likely not a factor in the increased RFE observed when a long pause or a slow stretch follow the active shortening contractions. Similarly, time alone is not sufficient for recovering full RFE following active muscle shortening, otherwise one could just wait sufficiently long in the force enhanced state (after the active muscle stretch) for RFE to recover fully. Rather it appears that following active shortening, the time prior to stretching or during stretching is important, not the time once stretching has been finished. In other words, the muscle must be primed for the stretching following an active shortening for full RFE to occur.

Another potential mechanism for the full recovery of RFE following the slow stretch (SSC_15°/s) or the long pause (SSC_1 s) conditions could be the recruitment of a passive elastic element upon activation, as first proposed by Edman *et al*. (1982). Edman speculated that in a pure stretch condition (RFE), a passive element “engages” upon muscle activation at the initial length. This passive element is then stretched, thereby contributing force beyond its contribution for a passively stretched muscle where the elastic element would not have engaged. During a shortening-stretch cycle, the engaged passive element is shortened (and thereby slackened), and quickly stretched back to its original length. Thus, the net elongation of the passive element is zero, and it does not contribute to the residual force enhancement, in agreement with our findings when little or no time is given between the shortening and stretch phases of our experiments, or when the muscle is stretched at a high speed immediately following the active shortening phase. However, if the slackened series elastic element following active muscle shortening can re-engage at the new (shortened) length given sufficient time, it could contribute to the RFE in a similar manner as if the muscle had been activated at the shortened length. Again, this potential mechanism is consistent with our observations that full RFE is re-established if muscle stretching follows the active shortening after a pause of about 1 s or more. Our results further suggest that this re-engagement of the passive elastic element can also occur during stretching, and thus, if the stretch speed is sufficiently slow and allows for sufficient time during the stretch, virtually the full RFE can be recovered. There has been much speculation about the possible passive element that might contribute to RFE, and might help explain the results found in this study. Recent experiments on the single myofibril and single sarcomere level, where structural, contractile, and regulatory proteins can be genetically or chemically eliminated and manipulated^24^,^25^,^26^,^27^, suggest that the filamentous protein titin is a prime candidate for explaining our results in a straightforward manner^5^,^28^. Nevertheless, it should be emphasized that although titin offers theoretically a beautiful solution for the observed results, titin’s role in RFE, and in the results observed in this study, is by no means established.

## Conclusion

We found that a one second interval between active muscle shortening and subsequent stretch abolished the depression of the residual force enhancement in human adductor pollicis muscle. Furthermore, we conclude that residual force enhancement preceded by shortening depends on the speed of stretch: increasingly slower stretch speeds generate increasingly more RFE. These results suggest that residual force enhancement preceded by an active shortening contraction is associated either with a depression of RFE because of an inhibition of cross-bridge attachment following shortening or a slackening of an elastic element (titin) upon active muscle shortening. This RFE depression can be recovered fully if there is sufficient time between the end of the active shortening and the end of the muscle stretch. Whatever the mechanism of recovery of the depressed RFE following active shortening, it must occur between the end of the shortening and the end of the stretching phase. Experiments in which the speed and magnitude of the active shortening, and the activation between the shortening and stretch phase are manipulated might provide additional insight if the RFE, and its recovery following active shortening, is primarily dependent on cross-bridges or passive structural elements.

## Additional Information

**How to cite this article**: Fortuna, R. *et al*. Residual force enhancement following shortening is speed-dependent. *Sci. Rep.* **6**, 21513; doi: 10.1038/srep21513 (2016).

## Figures and Tables

**Table 1:** Mean values of work, force before shortening (b-SHO), end of shortening (e-SHO) and end of stretching (e-STR) (±SE) for FD, SSC_0 s, SSC_0.5 s, SSC_1 s, and RFE for protocol 1.

	Work [J]		Force b-SHO [N]		Force e-SHO [N]		Force e-STR [N]	
Contraction condition	Mean	SD	Mean	SD	Mean	SD	Mean	SD
FD	1.65	0.26	58.2	8.0	20.2	4.3	–	–
SSC_1 s	1.60	0.19	59.5	6.5	18.1*	2.7	77.9	11.0
SSC_0.5 s	1.59	0.21	59.0	6.8	18.3	4.0	77.0	11.2
SSC_0 s	1.64	0.25	59.9	8.3	18.6	4.9	77.1	9.1
RFE	–	–	53.4	11.4	–	–	88.2†	14.9

^*^compared to FD.

^†^compared to SCC_1 s, SSC_0.5 s and SSC_0 s.

**Table 2:** Mean values of work, force before shortening (b-SHO), end of shortening (e-SHO) and end of stretching (−STR) (±SE) for FD, SSC_15°/s, SSC_60°/s, SSC_120°/s, and RFE for protocol 2.

	Work [J]		Force b-SHO [N]		Force e-SHO [N]		Force e-STR [N]	
Contraction condition	Mean	SD	Mean	SD	Mean	SD	Mean	SD264
FD	1.70	0.38	58.7	11.3	22.1	5.6	–	−265
SSC_15°/s	1.63	0.34	59.6	9.8	19.6	6.2	74.1	11.4
SSC_60°/s	1.65	0.40	59.6	9.4	19.8	6.8	73.5	10.8
SSC_120°/s	1.73	0.36	60.7	8.2	21.0	6.1	77.2*	9.9
RFE	–	–	56.5	17.4	–	–	82.8†	14.8

^†^compared to SCC_15°/s, SSC_60°/s and SSC_120°/s.

^*^compared to SSC_60°/s.
